# Enhancing quality of care for older adults: The age‐friendly health system (4Ms framework) and the role of general medicine physicians as system‐based complexologists

**DOI:** 10.1002/jgf2.748

**Published:** 2024-11-19

**Authors:** Masaya Higuchi, Takashi Watari, Kenya Ie

**Affiliations:** ^1^ Division of Palliative Care and Geriatric Medicine Massachusetts General Hospital Boston Massachusetts USA; ^2^ Harvard Medical School Boston Massachusetts USA; ^3^ Graduate School of Medicine Tohoku University Sendai Japan; ^4^ Integrated Clinical Education Center Kyoto University Hospital Kyoto Japan; ^5^ General Medicine Center Shimane University Hospital Shimane Japan; ^6^ Department of General Internal Medicine St. Marianna University School of Medicine Kanagawa Japan; ^7^ Department of General Internal Medicine Kawasaki Municipal Tama Hospital Kanagawa Japan

## Abstract

The aging population presents critical challenges to global healthcare systems, with Japan expected to have 35% of its population aged 65 or older by 2040. Older adults often experience multimorbidity, cognitive impairments, and physical frailties, increasing healthcare utilization and costs. Traditional medical approaches that focus on organ‐specific diagnoses are insufficient for addressing these multifaceted needs. The Age‐Friendly Health Systems (AFHS) framework, introduced in the U.S., offers a patient‐centered, value‐based approach to geriatric care, encompassing the "4Ms": Mentation, Mobility, Medication, and What Matters. These pillars prioritize cognitive health, physical function, appropriate medication use, and patient values. However, Japan has yet to implement this system widely. The integration of AFHS, with an additional focus on "Multicomplexity" (5Ms), aligns well with the core competencies of General and Family Medicine. This specialty is crucial in leading interdisciplinary efforts to enhance geriatric care, addressing fragmentation and variability in healthcare delivery. To successfully implement AFHS in Japan, general medicine physicians must be trained in managing complex conditions and coordinating care across specialties. This shift toward holistic, patientcentered care is essential for improving outcomes for older adults and reducing healthcare costs. Future research should focus on developing effective strategies for AFHS implementation and training healthcare teams for comprehensive care delivery.

## Graphical abstract

The diagram outlines the “Complexiologist Approach” within the 4Ms framework—Mentation, Mobility, Medication, and What Matters—highlighting the dynamic interplay between these domains in geriatric care. By continuously adjusting care strategies to the evolving needs of older adults, the Complexiologist ensures a holistic and integrated approach, optimizing patient outcomes in managing clinical complexity.

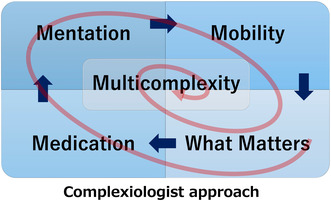

The rapid aging of the global population presents significant challenges to healthcare systems worldwide. According to the Ministry of Health, Labour and Welfare, by 2040, approximately 35% of Japan's population is projected to be aged 65 or older. Compared to other countries, Japan will have a particularly high proportion of elderly individuals, posing diverse challenges for healthcare providers in the care of older adults. This population often faces vulnerabilities such as multimorbidity, geriatric syndromes, and physical and cognitive impairments, which complicate the maintenance of independent living and negatively impact their quality of life. Consequently, there is an increase in healthcare utilization rates and costs compared to younger populations.^1^

While Japan has made significant progress in basic medical research, particularly in experimental fields, the traditional medical approach, which focuses on organ‐specific diagnosis and treatment, has proven inadequate in addressing the multifaceted needs of older adults and their care partners (CPs).^2^ To bridge this gap and effectively meet the needs of vulnerable older adults and their CPs, it is essential to implement Age‐Friendly Health Systems (AFHS) in Japan.^3^

This system aims to provide high‐value care that transcends beyond traditional disease‐centered approaches, emphasizing patient‐centered outcomes and improvements in quality of life (Figure 1). The AFHS framework is composed of four key elements (the 4Ms)^3^:

1. Mentation (Cognitive and Mental Health): Maintaining and improving cognitive function and mental health in older adults, including the early detection and treatment of dementia, as well as management and prevention of delirium and depression.
2. Mobility (Physical Function): Supporting independent living by maintaining physical function in older adults through fall prevention and rehabilitation.
3. Medication (Appropriate Prescribing): Ensuring appropriate prescriptions and medication management for older adults, including regular medication reviews to avoid potentially inappropriate medications, prevent drug‐related adverse events, and promote appropriate deprescribing to reduce the risks associated with polypharmacy (the use of multiple medications).
4. What Matters (Individual Values, Preferences, and Concerns): Respecting the individual values, life views, preferences, and concerns of older adults, thereby providing patient‐centered care.

**FIGURE 1:**
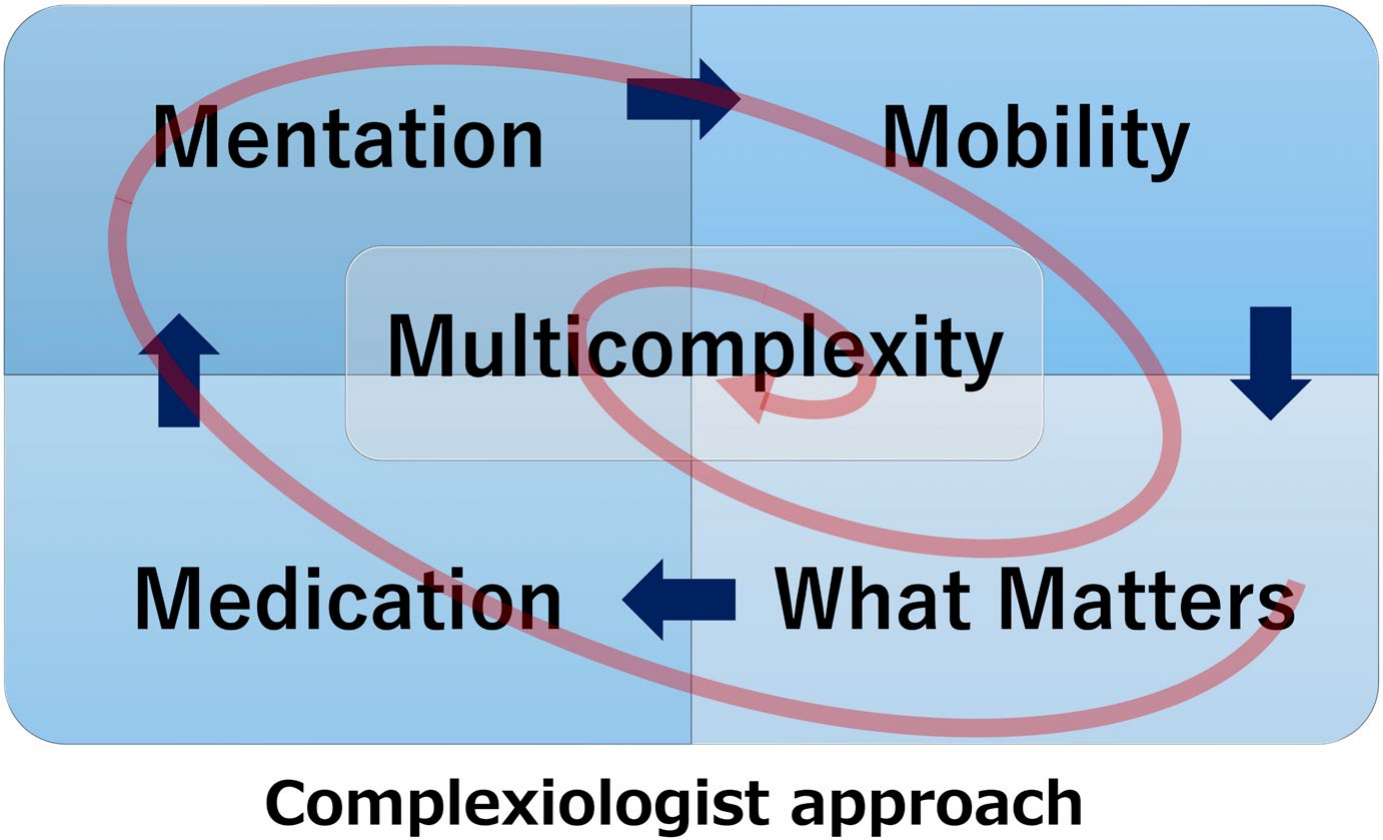
The Complexiologist Approach: Navigating Multicomplexity within the 4Ms Framework for an Age‐Friendly Health System. The diagram illustrates the “Complexiologist Approach” within the 4Ms framework—Mentation, Mobility, Medication, and What Matters. The arrows represent the dynamic and interconnected relationships among these domains in the care of older adults. The Complexiologist, a healthcare professional specializing in managing clinical complexity, employs a holistic and iterative approach, continuously adjusting care strategies in response to the evolving needs of the patient. This approach ensures that each domain is addressed in consideration of the others, thereby optimizing overall health outcomes for older adults with geriatric vulnerabilities.

Since 2018, more than 3922 healthcare institutions in the United States have implemented AFHS, providing evidence‐based geriatric care to over 3 million patients.^3^, ^4^ This has led to reported improvements in patient satisfaction, quality of care for older adults, and reductions in healthcare costs.^5^ However, in Japan, the core concept of system‐based care for older adults, as embodied in the AFHS, has yet to be sufficiently introduced or implemented. The transition to value‐based care, which emphasizes quality and cost, has not progressed, leading to a lack of an integrated approach and a comprehensive view of the overall healthcare system. Fragmentation, variability and the quality of care for older adults remain as significant issues. In Japan, interdisciplinary efforts and collaborations are crucial for advancing these improvements, with all clinicians, regardless of specialty, needing to be involved. Consequently, General and Family Medicine physicians, with their broad scope of expertise, undoubtedly play a key role in this initiative.

There are two main reasons for this. First, beyond the 4Ms, it is necessary to consider a fifth M, Multicomplexity (Figure 1).^5^ The 4Ms (Mentation, Mobility, Medication, What Matters) function interdependently, and managing these elements comprehensively in older adults requires not just skills, knowledge, and professional competence but also a sophisticated level of clinical acumen and intellectual rigor to address Multicomplexity in all its nuances and challenges.^3^, ^4^, ^5^

Second, the 5Ms framework, which is particularly necessary for implementing AFHS, significantly overlaps with the seven competencies emphasized in General and Family Medicine specialist training by the Japanese Medical Specialty Board. These competencies include the ability to (1) assess and manage complex situations, (2) integrate multiple health domains, (3) harmonize various treatment approaches, (4) orchestrate interdisciplinary healthcare teams, and (5) function effectively as practitioners of AFHS. These competencies are indispensable for effectively navigating the care of older adults within interdisciplinary teams, providing robust support to their CPs, and operationalizing age‐friendly systems to deliver high‐value, patient‐centered care. Among the 19 medical specialties in Japan, General and Family Medicine is the only specialty that explicitly identifies these skills as central to its training.

The AFHS framework represents a significant paradigm shift in system‐based geriatric care, offering a comprehensive approach to addressing the unique and complex needs of older adults. Given their vulnerability and the intricacies of their care requirements, this framework ensures a cohesive and holistic strategy across healthcare systems, effectively enhancing the quality of care provided to this population. The 5Ms framework serves as a comprehensive guide, empowering healthcare providers to deliver tailored, patient‐centered, effective, and high‐value care. It enables healthcare professionals to acknowledge and address the intricate needs and vulnerabilities of older adults through their expertise and skills. As the population continues to age, the importance and potential of AFHS will become increasingly significant. Future research should focus on refining implementation strategies, measuring outcomes, and training interdisciplinary healthcare professionals to excel within this new paradigm of care. In summary, in Japan, where the field of geriatrics is expected to play a pivotal role in the healthcare system, it is imperative to develop the ability to assess and manage complex medical situations, integrate multiple health domains, harmonize various treatment approaches, and lead team‐based care involving different specialists. These capabilities are essential for addressing the holistic needs of older adults and delivering patient‐centered care. Therefore, it is crucial that General and Family Medicine physicians spearhead the implementation of AFHS in Japan, ensuring that all healthcare professionals are equipped to meet the intricate and multifaceted demands of geriatric care.

## AUTHOR CONTRIBUTIONS

All authors had access to the utilized information and participated in the preparation of this manuscript.

## CONFLICT OF INTEREST STATEMENT

Takashi Watari and Kenya Ie is an Editorial Board member of Journal of General and Family Medicine and co‐authors of this article.

## CONSENT FOR PUBLICATION

All authors have provided their consent for publication.

## Data Availability

All relevant data have been included in the report.

## References

[1] Institute of Medicine (US) Committee on the Future Health Care Workforce for Older Americans . Retooling for an aging America: building the health care workforce. Washington (DC): National Academies Press (US); 2008 2, Health Status and Health Care Service Utilization. Available from: https://www.ncbi.nlm.nih.gov/books/NBK215400/ 25009893

[2] Watari T , Nakano Y , Gupta A , Kakehi M , Tokonami A , Tokuda Y . Research trends and impact factor on PubMed among general medicine physicians in Japan: a cross‐sectional bibliometric analysis. Int J Gen Med. 2022;15:7277–7285. 10.2147/IJGM.S378662 36133913 PMC9483137

[3] Mate K , Fulmer T , Pelton L , Berman A , Bonner A , Huang W , et al. Evidence for the 4Ms: interactions and outcomes across the care continuum. J Aging Health. 2021;33(7‐8):469–481. 10.1177/0898264321991658 33555233 PMC8236661

[4] John A . Hartford Foundation. Age‐Friendly Health Systems Initiative. Available from: https://www.johnahartford.org/grants‐strategy/current‐strategies/age‐friendly/age‐friendly‐health‐systems‐initiative [Accessed 4 Aug 2024].

[5] Holliday AM , Hawley CE , Schwartz AW . Geriatrics 5Ms pocket card for medical and dental students. J Am Geriatr Soc. 2019;67(12):e7–e9. 10.1111/jgs.16226 31802487 PMC9678016

